# Facile Preparation and Analytical Utility of ZnO/Date Palm Fiber Nanocomposites in Lead Removal from Environmental Water Samples

**DOI:** 10.3390/molecules27175592

**Published:** 2022-08-30

**Authors:** Basma G. Alhogbi, Ohowd Ibrahim, Mohamed Abdel Salam, Mohammed S. El-Shahawi, Mohammed Aslam

**Affiliations:** 1 Department of Chemistry, Faculty of Science, King Abdulaziz University, Jeddah 21589, Saudi Arabia; 2 Center of Excellence in Environmental Studies (CEES), King Abdulaziz University, Jeddah 21589, Saudi Arabia

**Keywords:** ZnO nanoparticles (NPs), nanocomposite solid-phase extractor, date palm fiber, Pb^2+^ removal, environmental water treatment

## Abstract

This study reports a facile approach for preparing low-cost, eco-friendly nanocomposites of ZnO nanoparticles (NPs) and date palm tree fiber (DPF) as a biomass sorbent. The hypothesis of this research work is the formation of an outstanding adsorbent based on the date palm fiber and ZnO nanoparticles. ZnO NP/DPF nanocomposites were synthesized by mixing the synthesized ZnO NPs and DPF in different mass ratios and evaluating their efficacy in adsorbing Pb^2+^ from aqueous solutions. The structure and surface morphology of the developed ZnO NP/DPF nanocomposite were critically characterized by XRD, FESEM, and TEM techniques. Compared to ZnO NPs, the ZnO NP/DPF nanocomposites displayed significantly enhanced Pb^2+^ uptake. Pb^2+^ adsorption was confirmed via various isotherm and kinetic models and thermodynamics. The computed Langmuir sorption capacity $(q_{m}$) was found to be 88.76 mg/g (R^2^ > 0.998), and the pseudo-second-order R^2^ > 0.999 model was most appropriate for describing Pb^2+^ adsorption. Impregnating the biomass with ZnO NPs enhanced the spontaneity of the process, and the value (−56.55 kJ/mol) of ΔH displayed the exothermic characteristics of Pb^2+^ retention. Only the loaded ZnO NP/DPF achieved the removal of a high percentage (84.92%) of Pb^2+^ from the environmental water sample (seawater). This finding suggests the use of ZnO NP/DPF nanocomposites for removing heavy metals from environmental water samples to purify the samples.

## 1. Introduction

Today, there is a lot of focus on modifying plant wastes to improve their surface and morphological properties before their application as solid-phase extractors (SPEs) for removing heavy metal ions from the aquatic environment. For example, three biosorbents of aquatic origin [1], palm tree waste fiber biosorbent [2], coffee husk biomass waste [3], raphia-microorganism composite biosorbent [4], and chemically modified plant wastes [5] were used to remove lead from aqueous solutions. A raw lignocellulosic biosorbent can be modified by physical treatment (using heat) or by chemical treatment (using acid) to increase acidic functional groups to enhance the biosorbent’s chelating ability with metal species, while treatment with a base enhances the uptake of organics [6,7]. The modification process improves the adsorbent’s affinity toward metal ion uptake and increases its adsorption capacity as the specific surface area and the number of adsorption sites increase. The distribution of the pore size is linked to the chemical structure of the solid-phase extractor, the biomass type of the raw material, and the synthesis of the raw material [8,9]. Chemical or physical modification is applied by loading the adsorbent surface with some donor atoms, such as oxygen, nitrogen, sulfur, and phosphorus, to bind with certain metal ions [10,11]. 

Nanotechnology offers new opportunities for wastewater treatment and provides innovative solutions to overcome the limitations and drawbacks of the conventional methods and the poor efficiency of removal of the most common organic and inorganic contaminants by treated biosorbents, such as plants [12,13,14]. Nanosized materials (size < 100 nm) comprise constituents with different chemical, biological and physical characteristics [14]. Nano-adsorbents show excellent performance in removing a series of inorganic and organic pollutants because of the large surface areas, physical forces, and/or weak chemical bonds of the organic pollutants [14,15]. The majority of the nanosized materials used for removing heavy metal ions from water are commonly divided into (i) organic nanomaterials, such as organic polymers and organic-polymer-supported nanomaterials, and (ii) inorganic nanomaterials, such as transition metal oxides [16]. The mechanisms nanomaterials use to remove heavy metal ions include adsorption, surface–metal complexation, ion exchange, electrostatic attraction, and acid–base interaction using processes such as film diffusion, diffusion of pores, and intra-particle diffusion separately or in combination [17]. 

Metal oxide nanoparticles, such as ZnO NPs, are characterized by excellent performance in removing heavy metal ions and oxyions from aqueous media [18]. These chemicals are characterized by an extraordinary surface area, tiny distance of intra-particle diffusion, and applicability without significant reduction in their surface area; and some of the nanosized metal oxides are superparamagnetic and can be separated and recovered via a low-gradient magnetic field [18]. ZnO is a multifunctional solid due to its unique features, e.g., excellent stability, extraordinary electrochemical properties, and good stability under light [18,19]. 

ZnO represents one of the most widely used nanosized materials, with numerous applications, for example, in sunscreens, in rubber, in dye material, as an animal feed ingredient, as antistatic pain additives, and in photo-catalysts [20]. ZnO NPs have a good porous structure that provides a high surface area and numerous surface-active sites [20], so ZnO NPs can be nominated as effective solid-phase microextractors for the complete removal of various pollutants from an aquatic environment [21]. Pollution by heavy metals is a major issue because of their toxicity to the aquatic environment. Recently, there has been an upsurge in interest in the use of solid waste, e.g., biomass, as a promising solid-phase extractor for a series of organic and inorganic pollutants from environmental water. In this study, the challenge is to provide cost-effective and effective solutions to environmental problems using solid wastes that are easy to handle, sensitive, and robust enough for field applications. The hypothesis of this research work is the formation of outstanding adsorbents based the date palm fiber and ZnO nanoparticles. Thus, this article is focused on (i) synthesizing and characterizing ZnO NPs and ZnO NP/DPF nanocomposites using XRD, TEM, and surface area analysis to explore their surface morphology, particle shape, size, and crystallinity, (ii) studying the impact of different analytical parameters, i.e., the solution pH, sorbent mass, contact time, Pb^2+^ ion concentration, and temperature that can enable controlling the removal of Pb^2+^ ions from aqueous media by the established ZnO Ns/DPF nanocomposites, (iii) assessing the kinetics, isotherm models, and the most probable mechanism of retention of Pb^2+^ from aqueous solutions, and (iv) testing the analytical utility of the established ZnO NP/DPF nanocomposites in removing Pb^2+^ ions from environmental water samples.

## 2. Results and Discussion

### 2.1. Characterization of ZnO NPs

**Figure 1:**
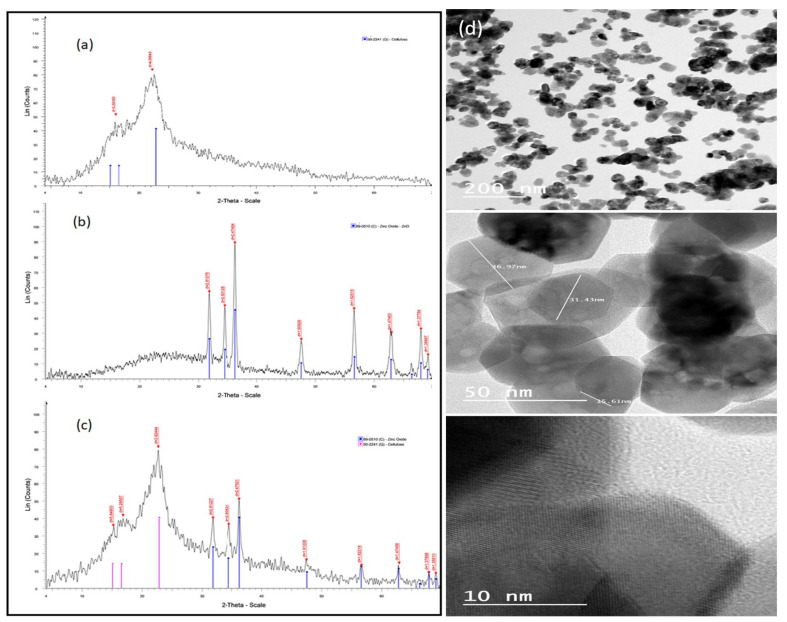
XRD patterns of the DPF sorbent (**a**), ZnO NPs (**b**), and the ZnO NP/DPF nanocomposite (**c**) and TEM images of ZnO NPs at different magnifications (**d**).

Figure 1 represents the XRD patterns of the natural date palm fiber sorbent (a), ZnO NPs (b), and the ZnO NP/DPF nanocomposite (c). The XRD pattern of ZnO NPs revealed three main characteristic diffraction peaks, at 2θ = 31.72, 34.38, and 36.20°, and these were safely assigned to the (100), (002), and (101) planes of hexagonal wurtzite ZnO, respectively. Accordingly, the XRD patterns of the ZnO NP/DPF nanocomposite had the characteristic peaks of both ZnO NPs and cellulose (JCPDS no. 89-0510, and JCPDS no 50-2241, respectively) [2].

**Figure 2:**
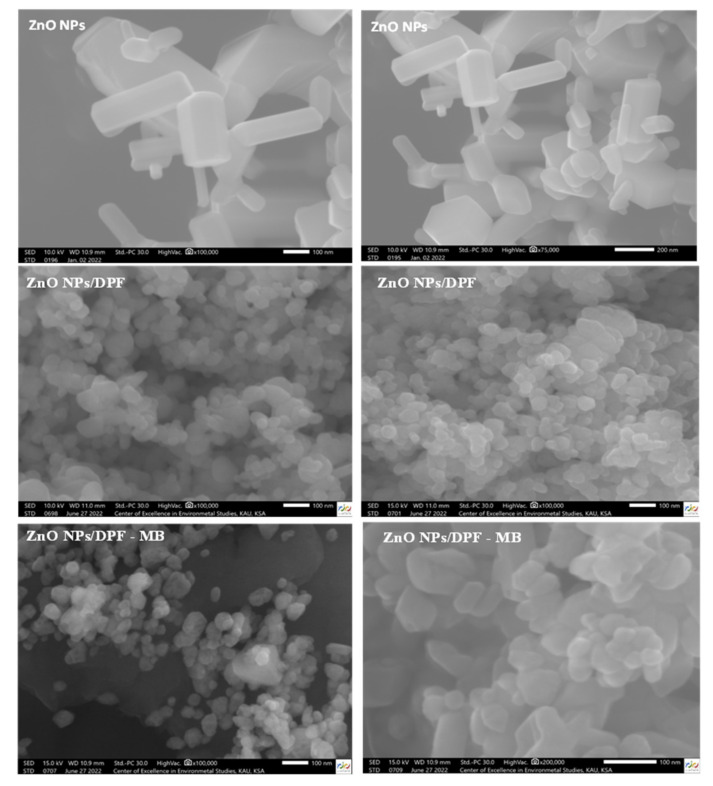
FE–SEM images of ZnO NPs, ZnO NP/DPF, and ZnO NP/DPF at different magnifications.

TEM measurements (Figure 1d) allow the characterization of the particle size, particle shape, and crystallinity of the ZnO NPs at different magnifications. The results revealed that ZnO NPs are agglomerated in a hexagonal shape with dissimilar lengths and a diameter of 30 nm. The FESEM images (Figure 2) of bare ZnO at different magnifications displayed a sharp-edged hexagonal ZnO particle with a wide distribution of particles 30–100 nm in size.

The Brunauer–Emmett–Teller (BET) N_2_ adsorption method was used to analyze the specific surface area and the total pore volume of the ZnO NPs, the DPF, and ZnO NP/DPF. The ZnO NPs had a BET specific surface area of 2.96 m^2^/g, with a total pore volume of 6.00 × 10^−3^ cm^3^/g, whereas the DPF had a BET specific surface area of 9.00 m^2^/g, with a total pore volume of 9.07 × 10^−3^ cm^3^/g. Upon the modification of the DPF and the formation of the ZnO NP/DPF, the BET specific surface area was greatly enhanced, to 15.8 m^2^/g, with a total pore volume of 2.62 × 10^−2^ cm^3^/g. This may be attributed to the electrostatic interaction between the lignocellulosic functional groups of the DPF [22] and the oxygen-containing functional groups at the ZnO NP surface [23,24].

### 2.2. Sorption Study

#### 2.2.1. Effect of Different Sorbent Ratio

**Figure 3:**
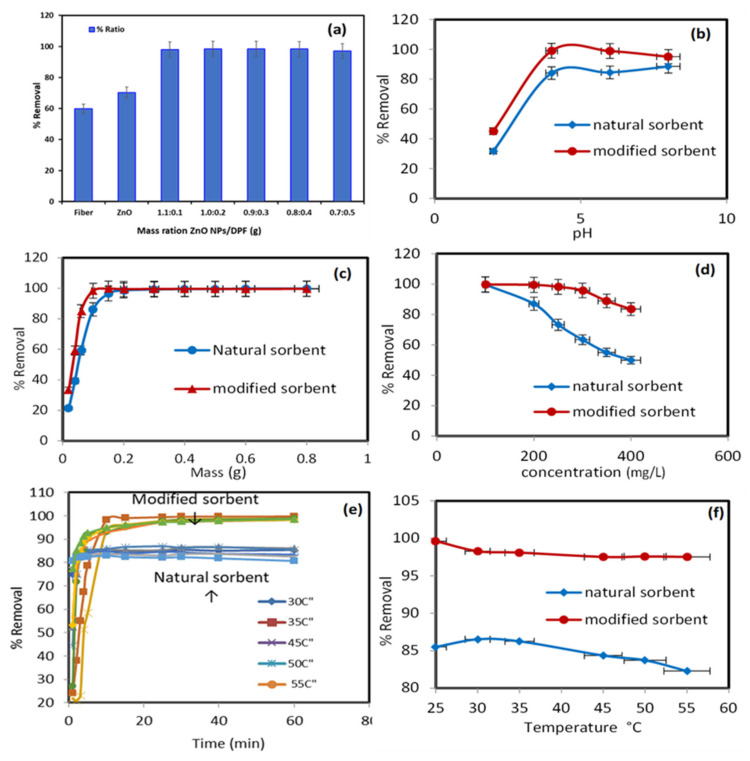
Impact of different parameters on the sorption percentage (%) of Pb^2+^ onto the modified sorbent ZnO NP/DPF: modified sorbent ratios (**a**), extraction media pH (**b**), sorbent dose (**c**), solution concentrations (**d**), contact time, and (**e**) and solution temperatures (**f**).

Figure 3 displays the percentage of Pb^2+^ ions extracted from the aqueous media by pure natural biosorbent DPF and modified DPF with ZnO NPs. The percentages of Pb^2+^ ion uptake by DPF and modified DPF with ZnO NPs were about 59.78% and 70.31%, respectively. However, different mass ratios of ZnO NP/DPF revealed a better extraction percentage of Pb^2+^, at around 98%. Therefore, the mass ratio of (1.1:0.1) of ZnO NP/DPF was selected in the experiments that followed.

#### 2.2.2. Influence of pH of the Extraction Medium 

The solution pH affected the sorbent surface charge. Thus, the uptake of Pb^2+^ (200 mg/L) by the tested solid sorbents was conducted under the following parameters: a wide range of pH (2 to 8), temperature = 25 °C, mass = 0.6 g, volume = 250 mL, and shaking time = 30 min. The data presented in Figure 3b show a low removal percentage for Pb^2+^ ions by the ZnO NP/DPF sorbent at pH 2 (adsorption capacity 15.3 mg/g) because of a protonated surface. At pH ≥ 4, however, the maximum adsorption percentage (99%) and an adsorption capacity 41.6 mg/g of Pb^2+^ were achieved by the modified DPT sorbent due to protonation of the modified and non-modified sorbent surface and increase in pH (to 4) due to deprotonation of the surface of both sorbents. Thus, the pH of the extraction media was adopted at pH 4 in the subsequent study. A similar trend was also reported for Pb^2+^ uptake using treated tea residue [25].

#### 2.2.3. Impact of Dosage of the Modified ZnO NP/DPF Sorbent

The resulting data of the effect of the ZnO NP/DPF sorbent with a modified mass are presented in Figure 3c. The parameters are: temperature = 25 °C, pH 4, concentration = 200 mg/L, volume = 250 mL, and shaking time = 30 min. The removal efficiency of Pb^2+^ ions increased from 33.47% to 99.70% with an increase in the ZnO NP/DPF mass from 0.02 g to 0.8 g at 30 min contact time. The Pb^2+^ ion removal efficiency increased from 21.38% to 99.8% with a growing DPF dose in the same interval. The modified mass of the ZnO NP/DPF sorbent used for the next experiment was 0.1 g, which obtained 98.4% removal.

#### 2.2.4. Effect of Lead (II) Concentration

The impact of the initial Pb^2+^ concentration (50–400 mg/L) on the ionic mass transfer between the aqueous solution with pH 4 and the solid phase of 0.1 g was studied at 25 °C. Figure 3d displays the obtained data. The percentage removal of Pb^2+^ by the ZnO NP/DPF and DPF sorbents decreased from 99.76% and 99.65% to 83.52% and 49.92% on increasing the initial Pb^2+^ ion concentrations from 50.0 to 400.0 mg/L, respectively. The decrease in the binding rate of Pb^2+^ onto modified and non-modified sorbent active sites most likely accounts for the observed trend. Thus, 200 mg/L was adopted as the optimum concentration of ZnO NP/DPF and DPF in the next experiment, where the percentage removal of Pb^2+^ ions was 86.99% and 99.4%, with adsorption capacities of 47.84 and 53.21 mg/g, using ZnO NP/DPF and DPF, respectively. Daneshfozoun et al. [26] studied the removal percentage of Pb^2+^ ions at the 200 mg/L initial concentration after 60 min of shaking time using raw oil palm empty fruit bunch (OPEFB). 

#### 2.2.5. Effect of Contact Time

The impact of the shaking time (5–60 min) on Pb^2+^ adsorption by the used solid sorbent was critically studied using the following parameters: Pb^2+^ ions = 200 mg/L, pH 4, sorbent mass = 0.6 g, volume = 250 mL, and a wide range of temperature (25–55 °C). The data presented in Figure 3e reveal that the percentage of Pb^2+^ ions removed by DPF increased rapidly within the first 10 min, reaching an equilibrium at 30 min, with a removal percentage of more than 80%, and the amount of Pb^2+^ adsorbed was 51.36 mg/g at 30 °C. The percentage of Pb^2+^ ions removed by ZnO NP/DPF increased rapidly within the first 5 min, until an equilibrium was reached after 10 min. In all, 90% of the Pb^2+^ ions were removed (the amount adsorbed was 86.16 mg/g at 30 °C). 

#### 2.2.6. Effect of Solution Temperature

The data in Figure 3f display the removal percentage of 200 mg/L Pb^2+^ ions by DPF and ZnO NP/DPF sorbents under the following parameters: pH 4, mass = 1.0 g, volume = 250 mL, and increasing solution temperature. On increasing the temperature of the extraction media from 25 to 55 °C, the uptake of Pb^2+^ decreased from 86.52% to 82.29% using DPF and from 98.28% to 97.52% using ZnO NP/DPF. Thus, an increase in temperature affects the interaction forces between sorbed Pb^2+^ ions and the available active sites of both the DPF natural sorbent and the modified ZnO NP/DPF sorbents. The data indicate the exothermic nature of the sorption process. 

### 2.3. Kinetic Study of Pb^2+^ Retention

**Figure 4:**
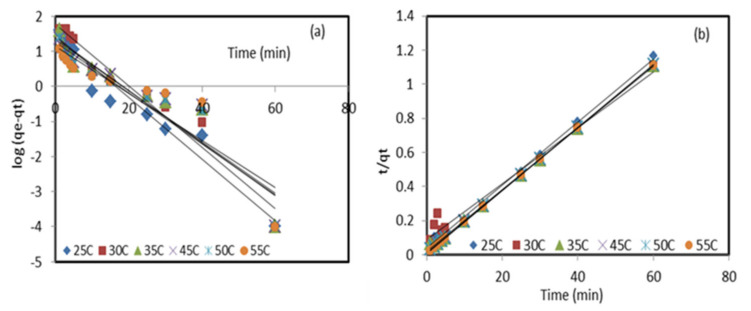
Pseudo-first-order (**a**) and pseudo-second-order (**b**) linear plots of Pb^2+^ retention by the modified sorbent ZnO NP/DPF at different temperatures.

To study the sorption mechanism of lead (II) ions by natural and modified sorbents, the data were critically subjected to the pseudo-first-order, pseudo-second-order [27], and intra-particle diffusion kinetic [28] models. The Lagergren model of the pseudo-first-order can be expressed by the following equation:

(1) $$Log\;\left(q_{e}-q_{t}\right)=log\;q_{e}-k_{1}t/2.303$$

where $q_{e}and\;q_{t}\;\left(\mathrm{mg}/g\right)$ are the adsorption capacities of Pb^2+^ ions at equilibrium and at time t, respectively, and $k_{1}\;\left(\min^{-1}\right)$ is pseudo-first-order adsorption rate constant. The $k_{1}$ and $q_{e}$ values were computed from the slope and the intercept of the linear plot of $log\left(q_{e}-q_{t}\right)versus\;t\;$ Figure 4. The pseudo-second-order kinetic used Equation (2):

(2) $$t/q_{t}=1/k₂q_{e}^{2}+t/q_{e}$$

where $k₂\;\left(g/\mathrm{mg}.\min\right)$ is the pseudo-second-order rate constant. The values of $q_{e}$ and k₂ are determined from the slope and the intercept, respectively, of the plotting of the values of $\frac{t}{q_{t}}$ versus time (Figure 4).

The adsorption kinetic model fit assessed by the correlation coefficient $\left(R^{2}\right)$ and the non-linear chi-square $\left(\chi^{2}\right)$ of the mathematical form of chi-square [29] is Equation (3):

(3) $$\chi^{2}={\left(q_{e,\;exp}-q_{e,\;cal}\right)}^{2}\;/\;q_{e,\;cal}$$

where $q_{e,\;exp}$ is the experimental equilibrium sorption capacity and $q_{e,\;cal}$ is the calculated sorption capacity. To understand the sorption mechanism of Pb^2+^ ions using natural and modified sorbents, the Weber–Morris model was adjusted to the experimental data attained and the Equation (4) was used:

(4) $$q_{t}=k_{int}\;t^{1/2}+C$$

where C is the thickness of the boundary layer and $k_{int}\;\left(\mathrm{mg}/g\cdot \min\right)$ is the intra-particle diffusion constant. The values of $k_{int}$ and C are determined from the slope and intercept of plotting $q_{t}\;\mathrm{vs}\cdot t^{1/2}\;$. Pseudo-first-order and pseudo-second-order kinetic models were applied to analyze the experimental data of Pb^2+^ ion adsorption onto the DPF natural sorbent and the modified ZnO NP/DPF sorbent, as reported in Table 1. The straight line of the experimental data represented in Figure 4b has an R^2^ value close to 1, and the calculated adsorption capacity *q_e,cal_* (55.183 mg/g) was quite close to the experimental *q_e,exp_* (54.028 mg/g), confirming the suitability of the pseudo-second-order kinetics in explaining the retention of lead (II) ions from the aqueous media at the optimized parameters by the modified sorbent. The χ^2^ values of the pseudo-second-order model were found to be below unity and smaller than those values obtained from pseudo-first-order presented in Figure 4a and Table 1. The results obtained from the removal of Pb^2+^ ions by the zeolite/ZnO nanocomposite for 50 mL of the solution with an initial concentration of 100 mg/L, pH 4, adsorbent dosage of 0.15 g, and contact time of 30 min followed pseudo-second-order sorption kinetics [30].

**Figure 5:**
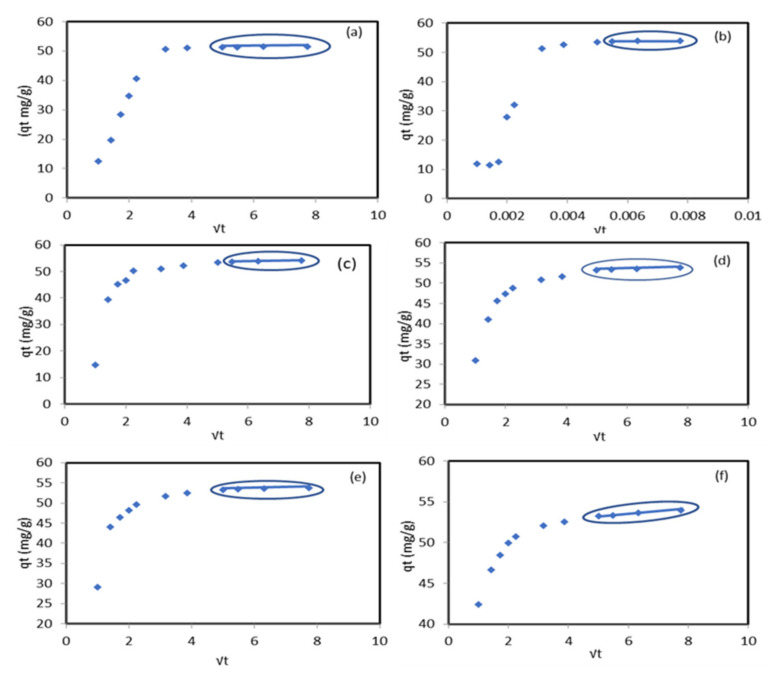
Weber–Morris intra-particle diffusion model of Pb^2+^ ion sorption onto the modified ZnO NP/DPF sorbent at different temperatures: 25 $℃\;\left(a\right)$, 30 $℃\;\left(b\right)$, 35 ℃ (**c**), 45 ℃ (**d**), 50 ℃ (**e**), and 55 ℃ (**f**).

The Weber–Morris intra-particle diffusion model was tested to assign the rate controlling step film or pore diffusion experimental data. The positive values of the boundary layer thickness C represented in Table 2 indicate the existence of the pore diffusion step. The plotting of $q_{t}\;vs.\;\surd t$ (Figure 5) shows that the multi-linear curve did not pass through the original two steps indicated in the sorption process. The first section is for Pb^2+^ diffusion onto the external surface, and the second section describes the diffusion inside the modified sorbent pores. The intra-particle diffusion was the rate-limiting step between different periods of time at each temperature as follows: (a) 25 ℃ between 30 min and 60 min, (b) 30 ℃ between 30 min and 60 min, (c) 35 ℃ between 25 min and 60 min, (d) 45 ℃ between 25 min and 60 min, (e) 50 ℃ between 25 min and 60 min, and (f) 55 ℃ between 25 min and 60 min. The authors of [31] indicated that the adsorption of Pb^2+^ ions by the Cu/ZnO nano-composite was controlled by both surface and intra-particle layer diffusion and the pseudo-second-order model was the best fit for the adsorption process.

### 2.4. Sorption Isotherm Study

To understand the equilibrium relationship between Pb^2+^ ions and the modified sorbent to obtain the maximum sorption capacity and percentage removal of Pb^2+^ ions the experimental data were subjected to four different adsorption isotherm models [32]. In the Langmuir model, the analyte uptake on a consistent surface is a monolayer sorption process without any interaction between adsorbed ions and is expressed by Equation (5):

(5) $$C_{e}\;/\;q_{e}=C_{e}/q_{m}+1/k_{L}q_{m}\;$$

where $C_{e}$ is the equilibrium concentration of the sorbate in solution (mg/L), $q_{e}$ is the equilibrium concentration of the sorbate on the adsorbent (mg/g), $q_{m}$ is the monolayer adsorption capacity (mg/g), and $k_{L}$ is the Langmuir adsorption constant (L/mg) determined from the slope and the intercept of the linear plot of $C_{e}/q_{e}\;vs.\;C_{e}$, respectively. The equilibrium parameter or separation factor $R_{L}$ is defined by Equation (6):

(6) $$R_{L}=1\;/\;\left(1+k_{L}C_{o}\right)$$

The Freundlich model is only applicable for a heterogeneous solid surface where the active sites have different energies, and the model can be expressed by Equation (7):

(7) $$logq_{e}=log\;k_{F}+1/n\;log\;C_{e}$$

where $k_{F}$ is the Freundlich constant of the relative adsorption capacity and 1/n is an empirical parameter and indicates the adsorption intensity or the surface heterogeneity.

The Temkin model assumes that the heat of adsorption of the analyte in the surface layer decreases linearly with increasing surface coverage because of adsorbent–adsorbate interactions. The Timken isotherm model can be expressed by Equations (8) and (9):

(8) $$q_{e}=\left(RT/b\right)\;ln\;k_{T}+\left(RT/b\right)\;ln\;C_{e}\;$$

(9) B=RT/b 

where *R* is the gas constant, B is a constant associated with the heat of analyte retention (kJ/mol), $k_{T}$ represents the Temkin isotherm constant (L/g), and b is the Temkin constant obtained from the slope and $k_{T}$ from the intercept of the plot $q_{e}$ vs. $ln\;C_{e}$.

The Dubinin–Radushkevich (D–R) model is an empirical model that describes adsorption onto a solid surface following a pore filling mechanism. It is generally applied to express the adsorption mechanism to distinguish the physical adsorption and chemical adsorption of metal ions by their mean free energy [33]. The linear equation of the D–R isotherm is expressed by Equation (10):

(10) $$ln\;q_{e}=ln\;q_{d}-\beta {ɛ}^{2}$$

where $q_{e}$ is the amount of adsorbed metal ions per unit weight of biomass (mol/L), $q_{d}$ is D–R isotherm constant related to the maximum biosorption capacity (mg/g), and β is the activity coefficient related to the biosorption mean free energy (mol^2^/J^2^). These parameters can be obtained from the plotting of $ln\;q_{e}$ versus ${ɛ}^{2}$ with slope β and intercept $ln\;q_{d}$. The Polanyi potential ɛ can be expressed by Equation (11):

(11) $$ɛ=RT\;ln\;\left(1+1/Ce\right)$$

The biosorption mean free energy E (kJ/mol) [34] is described as Equation (12):

(12) E=1 / √−2β

**Figure 6:**
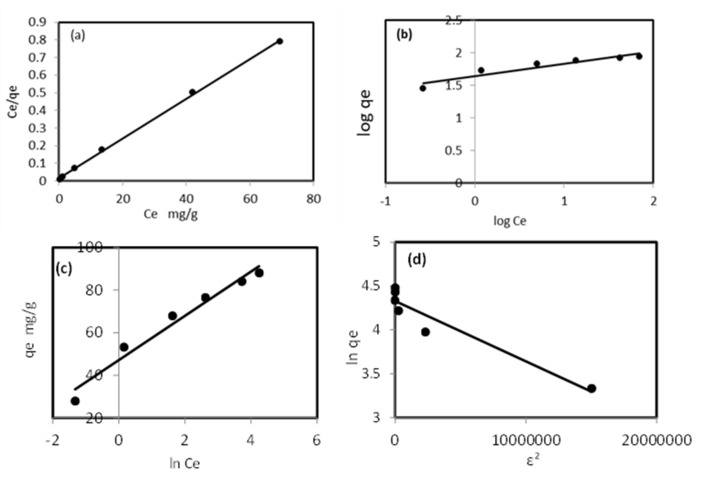
Langmuir (**a**), Freundlich (**b**), Temkin (**c**), and D–R (**d**) isotherm models for Pb^2+^ sorption by the developed modified ZnO NP/DPF solid extractor.

The studied isotherm models are illustrated in Figure 6, and the parameters are presented in Table 3. The Langmuir isotherm model of plotting the linear relationship *C_e_*/*q_e_* vs. *C_e_* is presented in Figure 6a, with a correlation coefficient (R^2^) of 0.9992 and a maximum sorption capacity $q_{m}$ of 88.76 mg/g (Table 3). These values indicate that the sorption of Pb^2+^ ions onto a modified sorbent is a good fit to the Langmuir isotherm model. The separation factor $R_{L}$ values ranged from 0.012 to 0.003, indicating that the modified sorbent was favorable for the removal of Pb^2+^ ions from the aqueous solution, forming a monolayer on the homogenous surface. The Freundlich isotherm model experimental data with a linear relationship are presented in Figure 6b. The sorption intensity 1/n value (0.19) lies between 0 and 1, and *n* (5.261) is less than 10, indicating that the sorption of Pb^2+^ was favorable but the process was a poor fit to the Freundlich model because R^2^ was 0.89. The data of the Temkin isotherm model are presented in Figure 6c, indicating that the sorption of Pb^2+^ ions onto the modified sorbent was a poor fit to the Temkin isotherm model, with an R^2^ of 0.939 and sorption heat β of 10.37 kJ/mol.

The Dubinin–Radushkevich isotherm model presented in Figure 6d was used for calculating the parameters given in Table 3. The value of free energy E was small (2.69 kJ/mol), and the removal of Pb^2+^ ions by the modified sorbent poorly fitted the D–R isotherm model, with R^2^ of 0.91, and most likely followed the pore filling mechanism onto the heterogeneous surface. The maximum adsorption capacity calculated by the Langmuir isotherm model for Pb^2+^ ion removal by both alkali (ATMS) and carboxyl (CFMS) functionalized Mangifera seed shell was 59.25 mg/g and 306.33 mg/g, respectively, and the D–R isotherm model indicated the mean free energy of 0.026 kJ/mol for the ATMS process and 0.137 kJ/mol for the CFMS process [35]. This may indicate the suitability of the Langmuir isotherm model compared with the other models, and the removal of Pb^2+^ ions from the aqueous solution, forming a monolayer on the homogenous surface of the modified ZnO NP/DPF sorbent.

### 2.5. Thermodynamic Study

In solid-phase extraction, the impact of the surface functional groups for the analyte uptake depends on the temperature of the extraction media. Thus, the adsorption capacity, the thermodynamic parameters involving activation enthalpy $\left(\Delta H\right)$, and the Gibbs free energy $\left(\Delta G\right)$ of the sorption step of the analyte are critical. These parameters were calculated using Equations (13)–(15):

(13) $$\Delta G=-RT\;ln\;k_{d}$$

(14) $$\ln k_{d}=-\frac{\Delta G}{R\;T}\;$$

(15) ΔG= ΔH –TΔS

where R is the ideal gas constant (8314 J/mol. K), T is the absolute temperature (*K*), and $k_{d}$ is the linear sorption distribution coefficient [36] defined by the Equation (16):

(16) $$k_{d}=q_{e}\;/\;C_{e}\;$$

where $q_{e}$ is the equilibrium quantity of the analyte adsorbed onto the adsorbent (mg/L) and $C_{e}$ is the equilibrium concentration of the adsorbate in the solution (mg/L). The values of ΔH and ΔS are determined from the slope and the intercept of the plot of ln kd versus 1/T. The numerical values of ΔH, ΔS, and ΔG are provided in Table 4. The value of ΔG confirmed the spontaneous nature of Pb^2+^ sorption onto the modified sorbent. The spontaneity of the Pb^2+^ sorption onto the modified sorbent decreased with increasing temperature. The value of ΔH (−56.5488 J/mol) indicated the exothermic nature of the retention step of lead ions. The electrostatic attraction and the negative value of ΔS indicated the decrease in the degree of freedom at the solid–liquid interface. The spontaneous nature of Pb^2+^ removal by ZnO nanoparticles is in good agreement with the data reported for ZnO prepared from the zerumbone and rhizomes in plant [37].

Based on the above results, the removal mechanism of Pb^2+^ by ZnO NP/DPF could be explained as follows: The electrostatic interaction between the lignocellulosic functional groups of the DPF [22] and the oxygen-containing functional groups at the ZnO NP surface [23,24] significantly enhance the BET surface area, which greatly enhances the removal efficiency of Pb^2+^ from aqueous solution. In addition, ZnO NP/DPF contains oxygen-based functional groups (carboxyl, hydroxyl, carbonyl, and lactone), which enhance Pb ions adsorption on the solid surface [38].

It is clear from Table 4 that, in comparison with different biosorbents, based on the removal capacity, ZnO NP/DPF is a competitive adsorbent for the removal of Pb^2+^ from an aqueous solution.

### 2.6. Environmental Application

**Figure 7:**
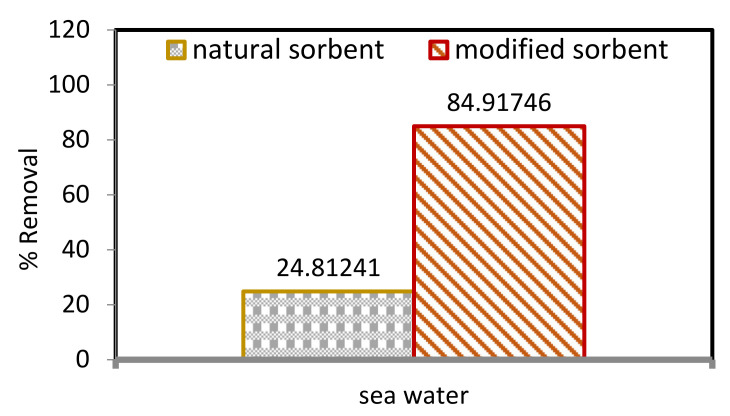
Comparison between natural and modified sorbents in removing Pb^2+^ ions from seawater.

Natural sorbents modified by ZnO NPs have improved efficiency in removing Pb^2+^ ions from seawater compared with DPF, as shown in Figure 7. Hence, the aim was to determine the Pb^2+^ concentration from the spiked solution of a 200 mg/L sample. The percentages of Pb^2+^ ions removed from seawater by DPF and ZnO NP/DPF sorbents were 24.81% and 84.92%, respectively. DPF and ZnO NP/DPF were found to be efficient in removing Pb^2+^ ions from seawater.

## 3. Materials and Methods

### 3.1. Synthesized ZnO NPs

All chemicals and reagents used were of analytical grade and used as received. ZnO NPs were prepared by apposite weight of zinc acetate dissolved in distilled water in the presence of 0.05% of Triton x-100 (0.05%). The solution mixture was heated to 80 °C under constant stirring for 4 h and subsequently hydrolyzed by dropwise addition of 0.2 M KOH (0.2M) until the solution pH was 8, with a hydrogel format. All the materials were supplied from BDH chemicals (GPR, England, 99%). The produced hydrogel was left overnight to achieve complete precipitation of the gel. The solution mixture was filtered, and the resultant precipitate was separated out, washed with water to remove the unreacted KOH, and rewashed with an ethanol/acetone (50:50) mixture. The resulting precipitate was allowed to dry overnight in a hot-air oven at 100 °C, dispersed, and calcined in a muffle furnace at 400 °C for 5 h.

### 3.2. Modified Palm Fiber Preparation

The ZnO nanoparticles were used to modify the date palm fiber (DPF) sorbent. In all, 1 g of the natural sorbent DPF (53 µm particle size) was physically grinded with 0.2 g of ZnO NPs. The effective ratio of the modified sorbent (0.1 g) was selected for different mixing ratios of ZnO NP/DPF, by weight in grams, ranging from 0.7:0.5 to 1.1:0.1, and shaken with 25 mL of 200 mg/L Pb(NO_3_)_2_ (Sigma-Aldrich, St. Louis, MI, USA, ≥99%) solution for 30 min. The solution mixture was filtered with Whatman filter paper (No. 2), and the Pb^2+^ concentration in the filtrate was measured using ICP-OES (Perkin Elmer Optima 8300 Inductively coupled Plasma-Optical emission spectrometry instrument). The impact of various analytical parameters, such as solution pH, concentration, mass, contact time, and temperatures, on the percentage of Pb^2+^ removed from the aqueous media by the ZnO NP/DPF nanocomposite were critically studied. The concentration of Pb^2+^ ions was measured by calculating the adsorption capacity and percentage removal using Equations (17) and (18).

(17) $$qe=\frac{\left(Co-Ce\right)}{m}\times V\;$$

(18) $$Removal\;\%=\frac{Co-Ce}{Co}\times 100\;$$

where *q_e_* is the amount of sorbed analyte (mg/g); *V* = solution volume (L); m = weight of the sorbent (g); and *C_o_* and *C_e_* are the initial and the equilibrium concentrations (mg/L), respectively. Note that all reported experimental data were based on the average of three replicates, accompanied by less than 5% error, indicating the homogeneity and stability of the nanocomposite from physical mixing of ZnO NPs and DPF under investigation. In addition, based on the experimental results, the hypothesis was validated as an outstanding adsorbent was prepared based on ZnO NPs and DPF.

### 3.3. Characterization of the Solid-Phase Extractor

The surface morphology of the modified and non-modified solid-phase extractor was performed using scanning electron microscopy (SEM, Quanta FEG 250 England) and JEOL-JEM-1230 transmission electron microscopy (TEM). Samples were suspended in ethyl alcohol and sonicated for 30 min, dried on a carbon-coated copper grid, and loaded into the TEM. A Philips X-pert pro diffractometer was used for recording X-ray diffraction (XRD) at 40 mA and 40 kV employing CuK α radiation and Ni filter in the range of 2θ between 2 to 80° in steps of 0.02°/s.

## 4. Conclusions

In this study, the removal of Pb^2+^ ions from an aqueous solution using non-modified natural date palm fiber and modified ZnO NPs was explored. ZnO NPs modified with DPF showed better efficiency in removing lead (II) than the non-modified biosorbent. The removal of Pb^2+^ ion increased rapidly within the first 5 min on increasing the dosage of both sorbents and reached an equilibrium at 30 min. The removal of Pb^2+^ by DPF and ZnO NP/DPF decreased with increasing temperature, indicating an exothermic sorption process. The sorption of Pb^2+^ ions onto DPF and ZnO NP/DPF fitted pseudo-second-order well. A Weber–Morris intra-particle diffusion plot indicated that both film and intra-particle diffusion controlled the sorption of Pb^2+^ ions onto the modified sorbent but the film diffusion was a rate-limiting step. The data of Pb^2+^ ion sorption fitted well with the Langmuir model, with a maximum monolayer sorption capacity of 88.76 mg/g. The Tempkin isotherm model revealed that the heat of sorption decreases with increasing monolayer coverage and the sorption of Pb^2+^ ions onto the modified sorbent poorly fits pore filling due to the small value of free energy obtained. Thermodynamic studies showed that the sorption process onto the ZnO NP/DPF modified sorbent was spontaneous due to the negative value of ΔG for all temperatures.

## Figures and Tables

**Table 1:** Pseudo-first- and second-order kinetic parameters of lead (II) retention by the modified sorbent ZnO NP/DPF at different temperatures.

Temperature °C	$q_{e,exp}$ $\left(mg/g\right)$	Pseudo-First-Order		Pseudo-Second-Order	
25	51.42	$q_{e,calc}\left(\mathrm{mg}/g\right)$	1.08	$q_{e,calc}\left(\mathrm{mg}/g\right)$	54.14
		R^2^	0.95	R^2^	0.99
		$k_{1}{\left(\min\right)}^{-1}$	0.20	$k_{2}$	0.11
		χ^2^	2358.26	χ^2^	0.14
30	54.02	$q_{e,calc}\left(\mathrm{mg}/g\right)$	5.83	$q_{e,calc}\left(\mathrm{mg}/g\right)$	60.99
		R^2^	0.96	R^2^	0.98
		$k_{1}{\left(\min\right)}^{-1}$	0.20	$k_{2}$	0.00
		χ^2^	398.51	χ^2^	0.80
35	54.03	$q_{e,calc}\left(\mathrm{mg}/g\right)$	3.99	$q_{e,calc}\left(\mathrm{mg}/g\right)$	55.18
		R^2^	0.90	R^2^	0.99
		$k_{1}{\left(\min\right)}^{-1}$	0.17	$k_{2}$	0.02
		χ^2^	627.04	χ^2^	0.02
45	53.80	$q_{e,calc}\left(\mathrm{mg}/g\right)$	3.81	$q_{e,calc}\left(\mathrm{mg}/g\right)$	54.41
		R^2^	$8.86e^{-1}$	R^2^	1.0
		$k_{1}{\left(\min\right)}^{-1}$	0.17	$k_{2}$	0.03
		χ^2^	656.99	χ^2^	0.01
50	53.80	$q_{e,calc}\left(mg/g\right)$	3.50	$q_{e,calc}\left(\mathrm{mg}/g\right)$	54.34
		R^2^	0.89	R^2^	1.0
		$k_{1}{\left(\min\right)}^{-1}$	0.17	$k_{2}$	0.03
		χ^2^	722.80	χ^2^	0.01
55	53.98	$q_{e,calc}\left(mg/g\right)$	3.09	$q_{e,calc}\left(\mathrm{mg}/g\right)$	54.20
		R^2^	0.83	R^2^	1.0
		$k_{1}{\left(\min\right)}^{-1}$	0.15	$k_{2}$	0.05
		χ^2^	836.86	χ^2^	0.00

**Table 2:** Intra-particle diffusion parameters for Pb^2+^ sorption onto the modified ZnO NP/DPF sorbent at different temperatures.

Temperature °C	$k_{int}\;\left(mg/g\;min\right)$	*C*
25	0.0266	51.221
30	0.1073	53.207
35	0.1945	52.541
45	0.1864	52.349
50	0.1773	52.439
55	0.2695	51.903

**Table 3:** Isotherm models and their parameters for Pb^2+^ sorption from model aqueous solutions onto the modified ZnO NP/DPF sorbent.

Isotherm Model	Parameter	Value
Langmuir	$q_{m}\;\left(\mathrm{mg}/g\right)$	88.76
	$k_{L}\;\left(L/g\right)$	0.76
	$R^{2}$	0.999
Freundlich	$k_{F}\;\left(\mathrm{mg}/g\right)$	43.76
	1n	0.190
	$R^{2}$	0.889
Temkin	$k_{T}\;\left(L/g\right)$	94.79
	$B\;\left(\mathrm{kJ}/\mathrm{mol}\right)$	10.37
	$R^{2}$	0.939
D–R	$q_{d}\;\left(\mathrm{mg}/g\right)$	75.89
	$E\;\left(\mathrm{kJ}/\mathrm{mol}\right)$	2.69
	$R^{2}$	0.912

**Table 4:** Comparison of the removal capacities of various biosorbents in terms of lead ions.

Biosorbent	Removal Capacity (mg/g)	Reference
**ZnO NP/DPF**	**51.42**	**Present study**
Green seaweed	2.25	[39]
Fish scales	24.26	[1]
Marine brown algae	64.5	[40]
DPF	39.50	[2]
Coffee husk biomass waste	19.07	[3]
Raphia-microorganism composite biosorbent	94.8	[4]
Aquatic plant	55.12	[41]
Cockle shell	24.66	[42]
Chitin of shrimp	7.00	[43]
Fish fins	3.00	[44]
Activated carbons (coconut shell)	32.08	[45]

## Data Availability

The authors confirm that data provide for the finding of this study are available inside the article. Row data that provide for the findings of this study are obtainable from the corresponding author, upon acceptable request.
